# Health Technology Assessment of Advanced Therapy Medicinal Products: Comparison Among 3 European Countries

**DOI:** 10.3389/fphar.2021.755052

**Published:** 2021-10-08

**Authors:** Lucia Gozzo, Giovanni Luca Romano, Francesca Romano, Serena Brancati, Laura Longo, Daniela Cristina Vitale, Filippo Drago

**Affiliations:** ^1^ Clinical Pharmacology Unit/Regional Pharmacovigilance Centre, University Hospital of Catania, Catania, Italy; ^2^ Department of Biomedical and Biotechnological Sciences, University of Catania, Catania, Italy; ^3^ Centre for Research and Consultancy in HTA and Drug Regulatory Affairs (CERD) University of Catania, Catania, Italy

**Keywords:** advanced medicinal products, health technology assessment, access, added therapeutic value, regulatory issue

## Abstract

Even for centrally approved products, each European country is responsible for the effective national market access. This step can result in inequalities in terms of access, due to different opinions about the therapeutic value assessed by health technology assessment (HTA) bodies. Advanced therapy medicinal products (ATMPs) represent a major issue with regard to the HTA in order to make them available at a national level. These products are based on genes, tissues, or cells, commonly developed as one-shot treatment for rare or ultrarare diseases and mandatorily authorized by the EMA with a central procedure. This study aims to provide a comparative analysis of HTA recommendations issued by European countries (France, Germany, and Italy) following EMA approval of ATMPs. We found a low rate of agreement on the therapeutic value (in particular the “*added value*” compared to the standard of care) of ATMPs. Despite the differences in terms of clinical assessment, the access has been usually guaranteed, even with different timing and limitations. In view of the importance of ATMPs as innovative therapies for unmet needs, it is crucial to understand and act on the causes of disagreement among the HTA. In addition, the adoption of the new EU regulation on HTA would be useful to reduce disparities of medicine’s assessment among European countries.

## Introduction

The centralized procedure adopted by the European Union (EU) and mandatory for some drugs category since 2004 (The Council of the European Communitie, 1993; European Parliament, Council of the European Union, 2001; European Parliament and the Council of the European Union, 2004) enables a rapid, EU-wide authorization of medicinal products based on a benefit/risk assessment, which requires the evaluation of quality, nonclinical, and clinical data on safety and efficacy submitted by the applicant. Once granted by the EU Commission, the centralized marketing authorization (MA) is valid in all member states. However, despite the unification of the procedures for drug approval, each country is responsible for an effective national market access, which needs the pricing and reimbursement decision to be adopted.

This can result in patient access inequalities among European countries, due to differences in terms of willingness to pay and opinion about the therapeutic value assessed by health technology assessment (HTA) bodies.

Generally, the HTA evaluates the added therapeutic benefits and risks for covering a new technology in the context of local standard of care (van Nooten et al., 2012), based on clinical (efficacy and safety), economic, ethical, and organizational aspects in support of policy decision-making about price and reimbursement decisions (Angelis et al., 2018). The heterogeneity of HTA recommendations, thereafter probably reflected in national reimbursement decisions and pricing agreements, is related to differences in assessment methodologies and health-care systems’ organization but also to the available evidence and, above all, willingness to accept uncertainty (Jommi et al., 2020).

Indeed, MA is increasingly granted by the European Medicines Agency (EMA) at earlier stages, especially for a high unmet medical need and/or rare diseases, through an accelerated assessment or conditional marketing authorization (CMA) before complete efficacy and safety data are available, thus potentially leading to limitations of evidence needed for the subsequent HTA process (Akehurst et al., 2017; Richardson and Schlander, 2019).

Acceleration of drug approval might therefore not always translate into positive and rapid patient access due to the uncertainties about the clinical benefits and the expected high impact on the health-care system (Ciani and Jommi, 2014; Akehurst et al., 2017; Allen et al., 2017).

Advanced therapy medicinal products (ATMPs) represent one of the clearest examples of issues for HTA (Drummond et al., 2019; Jönsson et al., 2019). These products are medicines for human use based on genes, tissues, or cells, commonly developed as one-shot treatment for rare or ultrarare diseases. The use of the centrally authorized procedure is compulsory for these innovative medicines. Due to the lack of well-designed clinical trials in terms of the number of patients enrolled, comparator, and long-term clinical data, frequently incomplete evidence is available to determine their value (Hettle et al., 2017).

The uncertainty is enhanced by their high cost which make the pricing and reimbursement decisions challenging (Garrison et al., 2019), with concerns about the affordability of health-care systems (Seoane-Vazquez et al., 2019). Moreover, even ATMP management and administration is complex and requires a clear definition of specialized centers and a proper funding (Ronco et al., 2021).

Several ATMPs have been licensed over the last decade, and the number of ATMPs reaching the market is expected to grow (Quinn et al., 2019), being hundreds in development, across numerous indications.

This study aimed to provide a comparative analysis of HTA recommendations issued by European countries following EMA approval of ATMPs.

## Methods

The study included the following steps:

1). Identification of approved advanced therapies in Europe between 2015 and 2020;
2). Identification of the reimbursement status and the HTA of ATMPs currently approved in Europe by the EMA performed by EU national authorities (France, Germany, and Italy); selection of countries was based on the availability of assessments for public consultation and on the clear definition of therapeutic values through comparable rating scales;
3). Comparative analysis of national opinions; available HTA reports and official administrative act of the three EU countries have been analyzed to compare ATMP assessments.

ATMPs centrally approved by the EMA have been identified by consulting the agency’s official documents and classified by type (gene therapy, cell therapy, and engineered tissues), according to the orphan drug designation, by type of authorization issued by the EMA (full, conditional, and for exceptional circumstances), and by therapeutic area (rare diseases, oncology, and others).

For each ATMP, pivotal clinical trials were reviewed, analyzing the study design, the number of patients enrolled, the primary and secondary outcomes, and the main study results.

The level of clinical benefit (*Service Médical Rendu*—SMR) and the added therapeutic value compared to the available therapeutic alternatives (*Amélioration du Service Médical Rendu*—ASMR) was extracted from the official HTA documentation resulting from the assessment of the Transparency Committee (TC) of the French National Authority (*Haute Autorité de santé*—HAS) (Santè, 2013; Santè, 2014).

With regard to Germany, we consulted the reports of the competent national bodies (*Federal Joint Committee* or *Gemeinsamer Bundesausschuss*, G-BA and *Institute for Quality and Efficiency in Health Care*, IQWIG) containing a complete HTA on the additional therapeutic benefit of the product compared to recognized standard therapies (Bundesausschuss, 2010).

Finally, we identified the therapeutic need, the added therapeutic value, and the quality of the evidence from the innovation assessment reports published by the Italian Medicines Agency (AIFA) (AIFA, 2017). A direct comparison among national opinions was possible in terms of an “added therapeutic value,” a measure included in all the available assessments (Supplementary Figure S1).

## Results

Currently, 12 ATMPs have been authorized in Europe (nine gene therapies, one cell therapy, and two engineered tissues) for 13 therapeutic indications between 2015 and 2020 (Supplementary Table S1). The MA for five additional products has been withdrawn. Ten out of 12 medicines received orphan designation, including three indicated for onco-hematologic diseases and five for genetic rare diseases. Tecartus^®^, Zolgensma^®^, Zynteglo^®^, and Holoclar^®^ received a conditional approval, whereas Tecartus^®^, Libmeldy^®^, and Zynteglo^®^ underwent an accelerated assessment (Table 1).

**TABLE 1:** ATMPs in Europe and their approval details.

*N*	Medicine name	Therapeutic area	Active substance	ATC code	Conditional approval	Exceptional circumstances	Accelerated assessment	Orphan medicine	Marketing authorization date
1	*Tecartus®*	Onco-hematologic	Brexucabtagene autoleucel	L01X	x	no	x	x	December 14, 2020
2	*Libmeldy®*	Rare diseases	Atidarsagene autotemcel	N07	no	no	x	x	December 17, 2020
3	*Zolgensma®*	Rare diseases	Onasemnogene abeparvovec	M09AX09	x	no	no	x	May 18, 2020
4	*Zynteglo®*	Rare diseases	Betibeglogene autotemcel	B06A	x	no	x	x	May 29, 2019
5	*Luxturna®*	Rare diseases	Voretigene neparvovec	Not assigned	no	no	no	x	November 22, 2018
6	*Yescarta®*	Onco-hematologic	Axicabtagene ciloleucel	L01X	no	no	no	x	August 23, 2018
7	*Kymriah®*	Onco-hematologic	Tisagenlecleucel	L01	no	no	no	x	August 22, 2018
8	*Alofisel®*	Other	Darvadstrocel	L04	no	no	no	x	March 23, 2018
9	*Spherox®*	Other	Spheroids of human autologous matrix-associated chondrocytes	M09AX02	no	no	no	no	July 10, 2017
10	*Strimvelis®*	Rare diseases	Autologous CD34 ^+^ enriched cell fraction that contains CD34 ^+^ cells transduced with retroviral vector that encodes for the human ADA cDNA sequence	L03	no	no	no	x	May 26, 2016
11	*Imlygic®*	Onco-hematologic	Talimogene laherparepvec	L01XX51	no	No	no	no	December 16, 2015
12	*Holoclar®*	Other	*Ex vivo* expanded autologous human corneal epithelial cells containing stem cells	S01XA19	x	No	no	x	February 17, 2015

In general, at the time of EMA approval, only data from open-label, single-arm, and phase I/II trials were available (Table 2), whereas data from concluded phase III, randomized, and controlled clinical trials were submitted for Luxturna^®^, Alofisel^®^, Spherox^®^, and Imlygic^®^. The median number of patients enrolled in these studies was 29 (range 1–436), followed for a median of 36 months (range 12–104).

**TABLE 2:** Data from clinical trials for ATMPs approved in Europe.

	Clinical trial	Study design	No. of patients	Primary outcome	Follow-up	Results
Tecartus® EMA CHMP (2020)	ZUMA-2	Phase II, single-arm, and open-label	74	Objective response rate (ORR)	36 months	ORR (85%) was significantly higher than the prespecified control rate of 25% at one-sided significance level of 0.025 (*p* < 0.0001)
Libmeldy® EMACHMP, n.d.	201222	Non-randomized, open-label, prospective, and comparative (nonconcurrent control)	22	Gross Motor Function Measure score (GMFM)	2–8 years long-term follow-up [median duration of posttreatment follow-up of 4.0 years (range: 0.6–7.5 years)]	An improvement of >10% of the total GMFM score in treated patients, when compared to the GMFM scores in the age-matched, untreated historical control MLD population, evaluated at Year 2 after treatment
Zolgensma^®^ EMA (2020)	CL-303	Phase III, open-label, single-arm	22	Event-free survival (event = death or permanent ventilation)	18 months	90.9% (95% CI: 79.7%, 100.0%) event-free survival at 14 months
	CL-101	Phase I, open-label, and dose-escalation	15	1. Requirement of respiratory assistance per day continuously for ≥2 weeks in the absence of an acute reversible illness, or 2. death.	24 months	All treated patients had statistically significant improved survival without permanent ventilation
		CL-302 (ongoing)	Phase III, open-label, and single-arm	33	Achievement of developmental milestone	18 months	The primary efficacy endpoint “independent sitting for at least 10 s at any time up to 18 months of age was met by 6 of the 32 patients (18.8%)
		CL-304 (ongoing)	Phase III, open-label, and single-arm	At least 44 (as of the DEC 31, 2019 data cutoff, 29 patients were enrolled)	Achievement of developmental milestone	As of the efficacy data cutoff date of DEC 31, 2019, patients in cohort 1 had been in the study for an average of 10.5 months (range: 5.1–18 months). Patients in cohort 2 had been in the study for an average of 8.74 months (range: 2–13.9 months)	All patients in the study were alive and free of permanent ventilation at the data cut-off
Zynteglo® EMA (2019)	HGB-204	Phase I/II, open-label, and single-arm	18	Proportion of patients who meet the definition of transfusion independence (TI)	Median (min, max) of 32.11 (23.1, 41.9) months	For patients of non-β0/β0 genotype treated in studies HGB-204 and HGB-205, 11 out of 14 patients (78.6%; 95% CI of 49.2–95.3%) met the definition of TI at any time. Only 2 out of 8 β0/β0 patients (25.0%; 95% CI of 3.2–65.1%) met the definition of TI at any time
	HGB-205	Phase I/II, open-label, and single-arm	4	Proportion of patients who meet the definition of transfusion independence (TI)	Median (min, max) of 38.29 (28.8, 47.7) months	
	HGB-207 (ongoing)	Phase III, open-label, and single-arm	23 (as of the February 22, 2018 data cutoff, 14 patients were enrolled and 10 treated)	Proportion of patients who meet the definition of transfusion independence (TI)	Median (min, max) of 5.59 (0.8, 13.2) months	As of February 22, 2018, follow-up time is limited and no patients had sufficient follow-up time that is needed for assessment of the primary endpoint
		HGB-212 (ongoing)	Phase III, open-label, and single-arm	1	Proportion of patients who meet the definition of transfusion independence (TI)	3.0 months	—
Luxturna® EMA (2018c)	301	Phase III, open-label, and randomized. placebo-controlled	31	Mean change from baseline to 1 year in binocular multi-luminance mobility testing (MLMT)	3 years	The monocular MLMT change score significantly improved in the treatment group and was similar to the binocular MLMT results
		101	Phase I, open-label, and dose-escalation	12	Safety	8 years	10 (83%) subjects experienced TEAEs considered related to the study drug administration e.g. eye irritation and hyperemia, one instance of macular hole
Yescarta® EMA (2018a)	ZUMA-1	Phase II, open-label, and single-arm	111	Objective response rate (ORR)	24 months: long-term follow-up of 15 years for those patients in response (ongoing)	ORR among all 101 subjects treated in phase 2 was 83% (95% CI: 74%, 90%), with a CR rate of 58%. ORR in all 111 enrolled patients in Cohorts 1 and 2 was 77% (95% CI: 69%, 85%) with a CR rate of 55% per local investigator
Kymriah®EMA (2018b)	B2202-ELIANA (ALL)	Phase II, open-label, and single-arm	97	Overall remission rate (ORR)	60 months: long-term follow-up of 15 years (ongoing)	The ORR was 82.7% (62/75) (95% CI: 72.2–90.4)
		C2201-JULIET (DLBCL)	Phase II, open-label, and single-arm	147	Overall response rate (ORR)	60 months: long-term follow-up of 15 years (ongoing)	Forty-three of 81 (53.1%) patients with at least 3 months follow-up demonstrated complete (32 patients; 39.5%) or partial (11 patients; 13.6%) response within 3 months after infusion
Alofisel® EMA CHMP (2017)	ADMIRE-CD	Phase III, randomized, double blind, parallel group, and placebo-controlled	212	Remission at Week 24 after study treatment	Long-term follow-up up to Week 52 (limited number of patients followed up to Week 104)	Statistically significant difference between the numbers of patients in combined remission in the active and placebo groups at week 24. The estimated difference between the groups was 15.2%
Spherox® EMA (2017)	Cod 16 HS 14	Phase II, open-label, and single-arm	75	Change of overall Knee Injury and Osteoarthritis Outcome Score	60 months	Statistically significant difference between the baseline value and posttreatment value
		Cod 16HS 13	Phase III, randomized, and open-label (comparator microfracture)	102	Change of overall Knee Injury and Osteoarthritis Outcome Score	60 months	Results are available from the Interim Analysis Report (12 months after treatment). The result of this interim analysis is that the ACT3D-CS treatment is not inferior to microfracture, but superiority was not demonstrated. Overall, KOOS at 12 months was however numerically in favor of ACT3D-CS
Strimvelis® EMA (2016)	AD1115611	Phase I/II, open-label, prospective, and sequential study	12	Survival	3 years	A 100% survival rate has been observed for all subjects (*N* = 18) who received GSK2696273 treatment in the pivotal and supportive studies and the CUP, with a median follow-up time of approximately 7 years
	AD1117056	Open-label, single-arm, and pilot study	2	Survival	3 years	
		AD1117054	Pilot study	1	Survival		13 years
		AD1117064	Compassionate use program (CUP)	3	Survival		3 years
Imlygic® EMA (2015)	Study 005/05	Phase III, randomized, and open-label (comparator GM-CSF)	436	Durable response rate (DRR)	36 months	The difference in DRR between Imlygic and GM-CSF in the ITT population was statistically significant in favor of Imlygic^®^
Holoclar® EMA (2014)	HLSTM01	Retrospective evaluation	106	Success of transplantation	Day 360: long-term follow-up up to 10 years	Treatment success was reported in 75 patients (72.1%) and failures were reported in 29 patients (27.9%), with an overall 95% confidence interval (CI) for success of 62.5–80.5% and a *p*-value < 0.001
		HLSTM02	Retrospective evaluation	29	Safety	Long-term data (>6 months) up to 8 years (most of the subjects were followed for at least 1 year)	Success according to the subjective, overall clinical judgment of the investigator, was reported in 19 out of 29 patients (65.5%) and failure in 6 patients (20.7%)

Table 3 reports the reimbursement status of ATMPs. Except for the latest ATMPs approved at the end of 2020, all ATMPs are reimbursed in at least one of the EU countries, and five out of 12 (41%) ATMPs are reimbursed in France, Germany, and Italy.

**TABLE 3:** Reimbursement status in France, Germany, and Italy of ATMPs approved by the EMA. X = reimbursed; /= not reimbursed or final opinion not available.

	Italy	France	Germany
Tecartus®	—	—	—
Libmeldy®	—	—	—
Zolgensma®	X^a^	X^b^	X
Zynteglo®	—	X^c^	X
Luxturna®	X^d^	X	X
Yescarta®	X^e^	X	X
Kymriah®	X^e^	X	X
Alofisel®	—	X^f^	X
Spherox®	—	—	X
Strimvelis®	X	—	—
Imlygic®	—	—	X
Holoclar®	X^g^	X^h^	X

a Patients weighing up to 13.5 kg and clinical diagnosis of SMA type 1 and onset of symptoms during the first six months of life, or genetic diagnosis of SMA type 1 (biallelic mutation in SMN1 gene and up to two copies of the SMN2 gene); AIFA registry mandatory to select eligible patients and to monitor treatment response, even for the management of risk-sharing agreement (payment at result).

b Recommendation for reimbursement in the treatment of patients with spinal muscular atrophy 5q (biallelic mutation of the SMN1 gene), with a clinical diagnosis of type I and II SMA or presymptomatic and having up to three copies of the SMN2 gene.

c Favorable opinion for reimbursement only in patients over 12 years to less than 35 years in the treatment of transfusion-dependent β-thalassemia (TDT), without β0/β0 genotype, eligible for hematopoietic stem cell transplantation (HSC), but not having related donor HLA compatible.

d AIFA registry mandatory to select eligible patients and to monitor treatment response.

e AIFA registry mandatory to select eligible patients and to monitor treatment response, even for the management of risk-sharing agreement (payment at result).

f In the sole treatment of uncomplicated complex perianal fistulas in adults with non-active/slightly active luminal Crohn’s disease.

g AIFA registry mandatory to select eligible patients and to monitor treatment response, even for the management of risk-sharing agreement (payment by results).

h In the treatment of patients with moderate to severe limbal stem cell deficiencies, caused by chemical or physical eye burns, who meet the following criteria: presence of a superficial corneal neovascularization in at least two quadrants of the cornea in at least one of the eyes and involvement of the central cornea and severely altered visual acuity.

A data analysis showed that for eight ATMPs, at least one public HTA evaluation from at least one of the three selected countries is available, and for five of these products, HTA reports have been published by all three countries (Table 4). At the time of the analysis, only the French authorities published opinion about the last ATMPs approved by the EMA in 2020, Tecartus^®^, and Libmeldy^®^.

**TABLE 4:** Agreement among opinions about therapeutically added value issued by member states.

	Italy	France	Germany
Tecartus®	—	III	—
Libmeldy®	—	III^a^	—
Zolgensma®	Important^b^	III/V^c^	—
Zynteglo®	—	III	Nonquantifiable
Luxturna®	Important	II	Considerable
Yescarta®	Important	III	Nonquantifiable
Kymriah®	Important	III^d^/IV^e^	Nonquantifiable^d^/-^e^
Alofisel®	Low	IV	Nonquantifiable
Spherox®	—	—	—
Strimvelis®	—	—	—
Imlygic®	—	—	Not proved
Holoclar®	—	IV	—

a Only in asymptomatic children without clinical manifestation of the disease.

b Patients weighing up to 13.5 kg and clinical diagnosis of SMA type 1 and onset of symptoms during the first six months of life, or genetic diagnosis of SMA type 1 (biallelic mutation in the SMN1 gene and up to two copies of the SMN2 gene).

c Patients with Type I SMA, presymptomatic with a genetic diagnosis of SMA (biallelic mutation of the SMN1 gene), and one to two copies of the SMN2 gene.

d Acute lymphoblastic leukemia.

e Diffuse large B-cell lymphoma.

An alignment of the three countries was identified in two out of five cases (16% of the total of 12 ATMPs). In particular, we found an agreement on the value of Luxturna^®^ (“*ASMR II*” for France; “*Significant additional advantage*” for Germany; and “*Important*” for Italy) and Alofisel^®^ (“*ASMR IV*” for France; “*Added benefit not quantifiable*” for Germany; and “*Poor*” for Italy). The assessments were, however, in disagreement for the following medicines: Zolgensma^®^ (“*ASMR III/IV*” for France; “*Evaluation not available*” for Germany; and “*Important*” for Italy), CAR-T Yescarta^®^ (“*ASMR III*” for France; “*Nonquantifiable*” for Germany; and “*Important*” for Italy), and Kymriah^®^ (“*ASMR III/IV*” for France; “*Nonquantifiable*” for Germany; and “*Important*” for Italy).

## Discussion

Patient access to new drugs requires MA from a regulatory authority and reimbursement by a payer. Despite the successful unification of the European procedures for drug approval, each country retains its own jurisdiction over the national market access, pricing, and reimbursement agreements adapted according to national health needs and health-care resources.

At a national level, policy-makers face several challenges when trying to design the most appropriate pharmaceutical policy measures, including the need for ensuring equitable and timely access.

The decision about reimbursement and national access usually needs an HTA appraisal, based on clinical, economic, ethical, and organizational elements. Universally recognized clinical criteria for HTA recommendations include unmet medical needs, relative effectiveness, and safety of the new product compared to the standard of care (if any) (van Nooten et al., 2012).

However, while relying on the evaluation of the same studies, a heterogeneity of the HTA of clinical data has been observed and may be reflected in disparity in terms of national reimbursement decisions and pricing agreements (e.g., coverage or not, treatment restrictions as regard to patients’ eligibility, and risk sharing agreement application).

In this context, a proposal for a regulation by the European Parliament and the Council on health technology assessment amending the Directive 2011/24/EU has been drafted in 2018 and modified in 2021, with the aim to ensure a permanent cooperation on HTA at the EU level, sharing joint clinical assessments, joint scientific consultations, horizon scanning, and voluntary cooperation in nonclinical areas (European Commission, Directorate-General for Health and Food Safety, n.d.).

Advanced therapies represent an important innovation in the treatment of unmet medical needs, which allows to act on the primary cause of a disease with the possibility of complete recovery in many cases. Indeed, ATMPs may provide significant health benefits generally with a single administration, improving patient outcomes potentially over the long term.

However, due to the high level of both clinical and economic uncertainties, and the need for ensuring a rapid access to a possible curative therapy in patients with no adequate treatment options, ATMPs represent a major issue with regard to the assessment of their value in order to make them available at the national level.

This study shows a low rate of agreement on the therapeutic value (in particular the “*added value*” compared to the standard of care) of ATMPs approved in Europe to date, having been found only in two cases out of 12 products authorized by the EMA (16%) and out of five ATMPs for which opinions are available in the three countries selected for the analysis (40%). This difference was not related to the choice of different comparators by HTA bodies, due to the lack of alternative therapies in most of the indications, or the availability of one single comparator.

The type of EMA authorization seems not to correlate with the national opinion. CMA, granted before complete efficacy and safety data are available, could potentially lead to limitations of the HTA process. However, even ATMPs approved with CMA (e.g., Tecartus^®^, Zolgensma^®^, and Zynteglo^®^) obtained positive assessment (“important” or “moderate”) with regard to the therapeutic added value.

The assessments issued by AIFA were particularly positive, since the added therapeutic value has been classified as “*important*” in four cases out of 12 (33%), corresponding to four over five ATMPs for which the evaluation has been made public (80%). On the other hand, the French and German authorities granted an “*ASMR II*” and a “*considerable additional advantage*,” respectively, only in the case of one ATMP (Luxturna^®^) out of 12 (8%), corresponding to one over seven (14%) and one over five ATMPs for which the assessment has been made public to date (20%). This is probably due to the particular attention to rare and ultrarare diseases given by the Italian agency, which explicitly accepts the possibility of having a low quality of evidence in these cases (AIFA, 2017). This results in a different impact on the general value given above all to the lack of a direct comparison with available treatment options, and the low number of patients enrolled. For example, a disagreement has been observed for Zolgensma^®^ assessment, with a score “*important*” for Italy, “*ASMR III/V*” for France, and no opinion for Germany. The dossier submitted by the company for MA included two complete trials (CL-303 and CL-101), both open-label and single-arm, and conducted on a few dozen patients (22 and 15, respectively). Even considering all the limitations of the indirect comparison, the Italian report highlighted that patients treated with Zolgensma^®^ obtained better clinical outcome than those treated with the antisense oligonucleotide nusinersen, the only drug available to date (AIFA, 2021). On the contrary, the French institutions considered that the lack of a direct comparison in clinical trials did not allow to define the place in therapy of Zolgensma^®^ with respect to nusinersen (Santè, 2020). Moreover, uncertainties have been raised about maintaining the effect of the treatment. Indeed, long-term data, both with regard to safety and effectiveness, are considered essential to determine the true therapeutic value of these products. The study plan of all ATMPs includes long-term data collection in line with regulatory requirements (The European Parliament and the Council of the European Union, 2007; CHMP CfMPfHU, 2018), but this follow-up is usually still ongoing.

Finally, the German G-BA delayed the assessment of the added therapeutic value of Zolgensma^®^ due to the limited clinical data available so far, and for the first time mandated a company to collect real-world evidence through a registry study in order to close the evidence gaps (Bundesausschuss, 2021). In particular, the G-BA expects a direct comparison of Zolgensma® with nusinersen, and any doctors who want to use the ATMP must take part in the study.

It is noteworthy that on March 2021, the EMA approved a new molecule for the treatment of patients with spinal muscular atrophy, risdiplam, which acts as a splicing modifier that increases and maintains the level of functional protein (EMA Evrysdi, 2021). Even with no direct comparison, it demonstrated a better efficacy and safety profile than nusinersen, as well as an important advantage of administration (oral vs. intrathecal).

Therefore, the availability of the new drug will probably increase the complexity in assessing the therapeutic value of Zolgensma^®^, also taking into account the high cost of the advanced therapy which has been defined as the most expensive ever.

In France, medicines can be reimbursed only in case the *Service Médical Rendu* (SMR, absolute clinical value) assessed by the TC of the HAS and is considered sufficient for all ATMPs obtained “*important*” as the SMR value for the reimbursed indication (Table 3).

However, patient treatment is possible also before the MA (and/or before the decision on the reimbursement of the authorized products), thanks to the Authorization for Temporary Use (ATU; Nominative or Cohort ATU), which allows early access for those with serious or rare diseases without other therapeutic options (ANSM, 2021). Actually, these schemes have been used for targeted therapies, immunotherapy, and ATMPs. For example, Luxturna^®^, Kymriah^®^, Yescarta^®^, and Zolgensma^®^ have been used in France according to the ATU scheme (ANSM, 2019a; ANSM, 2019b; ANSM ATU, 2018; ANSM, 2021).

During the ATU validity, the company can set a free price before the negotiation, but subsequently, the ASMR will be a driver for price negotiation.

In Italy, the AIFA Scientifc Technical Committee (Commissione Tecnico Scientifca, CTS) establishes the reimbursement status of new drugs according to the clinical added value and their place in therapy. In the case of positive opinion, the Price and Reimbursement Committee (Comitato Prezzi e Rimborso, CPR) negotiates with the company the price and any reimbursement agreements.

Moreover, the Law 648/1996 ensures reimbursement and nationwide access to innovative medicinal products authorized in foreign countries but not in Italy, to medicinal products not yet authorized but under clinical trial, and to off-label uses (648 L. Conversione in legge del decreto-legge 21 ottobre 1996 n, 1996; Gozzo et al., 2020a; Gozzo et al., 2020b; Brancati et al., 2021a; Brancati et al., 2021b). For example, the use of Zolgesma^®^ has been granted, thanks to this Italian law before the AIFA reimbursement agreement [(X). Inserimento; (X). Regime di rim].

In Germany, not all ATMPs are assessed as medicines. First of all, the G-BA must categorize the ATMPs as medicines or as medical procedures; subsequently, if considered as medicine, the benefit assessment procedure will be performed. Excluding Spherox^®^ and Holoclar^®^, all ATMPs have been classified as medicines, and their relative prices have been negotiated taking into account their added therapeutic value (Theidel U von der Schulenburg JM, 2016; Templin et al., 2019; Ronco et al., 2021). Despite the differences in terms of assessment, the access has been usually guaranteed (five over 12 products reimbursed in the three countries at the time of the analysis), even if with various timing and type of restrictions and even with the consequent sustainability issues. This is probably linked to the seriousness of the diseases and/or to the availability of other effective therapeutic treatments. In particular, excluding Tecartus^®^ and Libmeldy^®^, the two ATMPs most recently approved by the EMA, the three not equally reimbursed are indicated for not life-threatening diseases (cartilage defects or perianal fistulas) or for diseases with other treatment options available (melanoma).

The main limitation of the study is that results are not generalizable to all EU member states. However, countries selected for the analysis are those with a long experience in the HTA and with a clear definition of the therapeutic value through rating scales.

In view of the importance of ATMPs for the treatment of rare and serious unmet needs, it is crucial to understand and act on the causes of disagreement among the HTA, in order to ensure rapid and uniform access to these innovative therapies for all patients eligible for treatment. The adoption of the new regulation on HTA would be useful to harmonize HTA methodologies, hopefully leading to reduced disparities of medicines assessment among European countries.

## Data Availability

The raw data supporting the conclusion of this article will be made available by the authors, without undue reservation.
