# Supplementary material for: Health Technology Assessment of Advanced Therapy Medicinal Products: Comparison Among 3 European Countries
Source: Front Pharmacol. 2021 Oct 8;12:755052. doi: 10.3389/fphar.2021.755052 (PMC8531540; doi:10.3389/fphar.2021.755052)

**Supplementary table 1.** ATMPs approved in Europe and their therapeutic indications

| **N** | **Product** | **Type** | **Therapeutic indication** |
| --- | --- | --- | --- |
| ***1*** | ***Tecartus®*** | Gene | Treatment of adult patients with relapsed or refractory mantle cell lymphoma (MCL) after two or more lines of systemic therapy including a Bruton’s tyrosine kinase (BTK) inhibitor. |
| ***2*** | ***Libmeldy®*** | Gene | Treatment of metachromatic leukodystrophy (MLD) characterized by biallelic mutations in the arylsulfatase A (ARSA) gene leading to a reduction of the ARSA enzymatic activity:  - in children with late infantile or early juvenile forms, without clinical manifestations of the disease,  - in children with the early juvenile form, with early clinical manifestations of the disease, who still have the ability to walk independently and before the onset of cognitive decline |
| ***3*** | ***Zolgensma®*** | Gene | Patients with 5q spinal muscular atrophy (SMA) with a bi-allelic mutation in the SMN1 gene and a clinical diagnosis of SMA Type 1, or with 5q SMA with a bi-allelic mutation in the SMN1 gene and up to 3 copies of the SMN2 gene. |
| ***4*** | ***Zynteglo®*** | Gene | Patients 12 years and older with transfusion-dependent β thalassaemia (TDT) who do not have a β0/β0 genotype, for whom haematopoietic stem cell (HSC) transplantation is appropriate but a human leukocyte antigen (HLA)-matched related HSC donor is not available. |
| ***5*** | ***Luxturna®*** | Gene | Treatment of adult and paediatric patients with vision loss due to inherited retinal dystrophy caused by confirmed biallelic RPE65 mutations and who have sufficient viable retinal cells. |
| ***6*** | ***Yescarta®*** | Gene | Adult patients with relapsed or refractory diffuse large B-cell lymphoma (DLBCL) and primary mediastinal large B-cell lymphoma (PMBCL), after two or more lines of systemic therapy. |
| ***7*** | ***Kymriah®*** | Gene | Paediatric and young adult patients up to 25 years of age with B-cell acute lymphoblastic leukaemia (ALL) that is refractory, in relapse post-transplant or in second or later relapse.  Adult patients with relapsed or refractory diffuse large B-cell lymphoma (DLBCL) after two or more lines of systemic therapy. |
| ***8*** | ***Alofisel®*** | Cell | Complex perianal fistulas in adult patients with non-active/mildly active luminal Crohn’s disease, when fistulas have shown an inadequate response to at least one conventional or biologic therapy. |
| ***9*** | ***Spherox®*** | Tissue | Repair of symptomatic articular cartilage defects of the femoral condyle and the patella of the with defect sizes up to 10 cm^2^ in adults. |
| ***10*** | ***Strimvelis®*** | Gene | Patients with severe combined immunodeficiency due to adenosine deaminase deficiency (ADA-SCID), for whom no suitable human leukocyte antigen (HLA)-matched related stem cell donor is available |
| ***11*** | ***Imlygic®*** | Gene | Treatment of adults with unresectable melanoma that is regionally or distantly metastatic (Stage IIIB, IIIC and IVM1a) with no bone, brain, lung or other visceral disease |
| ***12*** | ***Holoclar®*** | Tissue | Patients with moderate to severe limbal stem cell deficiency, unilateral or bilateral, due to physical or chemical ocular burns. |

**Supplementary figure 1.** *Grading of added therapeutic value according to national opinions*


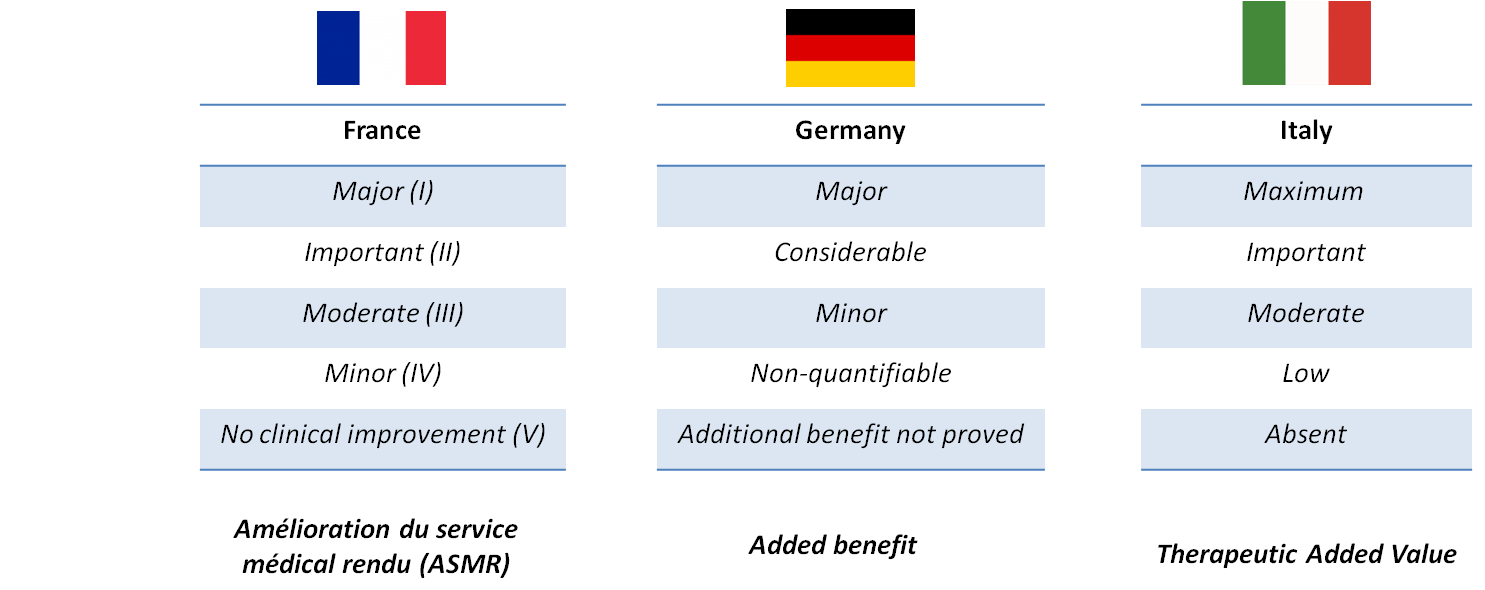

Supplement: Supplementary file 1 [file DataSheet1.docx]
